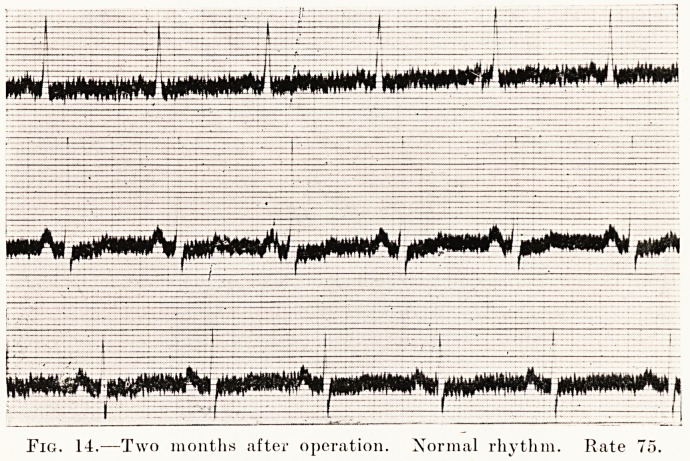# The Treatment of Graves' Disease
*Paper read at a meeting of the Bath and Bristol Branch of the British Medical Association, 28th May, 1930.


**Published:** 1930

**Authors:** C. E. K. Herapath

**Affiliations:** Physician, Bristol Royal Infirmary and Winford Hospital


### THE TREATMENT OF GRAVES' DISEASE.*

BY

C. E. K. Herapath, M.C., M.D.,

*Physician, Bristol Royal Infirmary and Winford Hospital.*

---

Five years ago I was of the opinion that the treatment of Graves' disease by operation was barely justifiable, but at the same time I was profoundly disappointed with the results of treatment by medical means. All the usual remedies had been tried—rest in bed for long periods, bromides, belladonna, sodium phosphate, iodine in large doses, and in small doses very frequently repeated, pancreas, insulin and deep X-rays—but the results were poor. In many cases there was improvement for a time, but the cases usually relapsed after a while, and the ground gained was lost in a short period; in other cases medical treatment seemed to do no good at all. The eradication of septic foci has long been advised and has been tried. For instance, in one case, that of a woman with pronounced Graves' disease, the teeth were very septic and a dentist was asked to clear the mouth. This was followed by a marked improvement, the thyroid became almost normal in size, the tachycardia quieted down and she gained a stone in weight. Six months later she returned to out-patients, and was just as bad as she was when first seen. Graves' disease is one of those conditions which may have marked remissions, and very often a remission has been taken to be the result of treatment.

When Mr. Short began his work on goitres he kindly asked me to see all his cases at the Bristol

* Paper read at a meeting of the Bath and Bristol Branch of the British Medical Association, 28th May, 1930.

Royal Infirmary, so that I might watch the results of operation. It was my custom to see them before and then again after the operation ; and if this was done in more than one stage, I saw them after each stage.

It is obvious that one of the best methods of demonstrating the effects of treatment is by an electrocardiogram. Lead 1 is taken from the two hands, and therefore shows the amount of tremor present. The amount of tachycardia is well seen in all leads, and any irregularity of the heart such as auricular fibrillation can be shown. Willius, in analysing 500 consecutive cases of this irregularity, found that Graves' disease was responsible for 27·9 per cent. and toxic adenoma of the thyroid for 20·5 per cent. of cases, which shows what an important factor the thyroid is in the production of auricular fibrillation. Records of 45 cases have been taken, but as only a few cases can be quoted here, I have selected 5 to show various points.

**Case 1.**—S. B., a woman with severe Graves' disease, had a marked tremor and a pulse-rate of 141. (Fig. 1.) Six months after operation she showed practically no tremor, and her pulse-rate had come down to 85. (Fig. 2.)

**Case 2.**—E. C., a woman who had had Graves' disease for eleven years, had been treated by me for three years with no improvement. At the end of this time she had tremor, tachycardia of 140, marked emaciation, and she was a nervous wreck. She was operated upon in two stages, and after six months the tremor was almost gone, her pulse-rate was 93, her nervousness was much improved, and she had put on a lot of weight ; in fact, when she came to see me I did not recognize her ; she looked a different woman. (Figs. 3 and 4.)

**Case 3.**—G. G. was a man who had been treated for twelve months in Australia for Graves' disease. They had tried every method of treatment, short of operation, and in the end they told him that they could do nothing more for him, so he returned to England. When I saw him he was a very

### PLATE V.

Fig. 1 (Case 1).—Marked tremor. Pulse rate 141. R's very high.

Fig. 2 (Case 1).—Six months after operation, tremor slight. Pulse rate 85. R's much smaller.

Fig. 3 (Case 2).—Pulse rate 140. Tremor marked. R's high.

### PLATE VI.

Fig. 4 (Case 2).—Pulse rate 93. Six months after operation. R's smaller. Lead III., altered.

Fig. 5 (Case 3).—Transverse diameter 16·1 cm. 30th January, 1928.

Fig. 6 (Case 3).—Transverse diameter 14·7 cm. 25th June, 1928.

### PLATE VII.

Fig. 7 (Case 3).—Pulse rate 153.

Fig. 8 (Case 4).—Five months after operation. Pulse rate 109.

Fig. 9 (Case 4).—Auricular fibrillation, slowed by digitalis.

### PLATE VIII.

FIG. 10 (Case 4).—Three weeks after operation, normal rhythm.

FIG. 11 (Case 5).—Transverse diameter 15·7 cm. 16th September, 1929.

FIG. 12 (Case 5).—Transverse diameter 13·5 cm. 15th November, 1929.

PLATE IX.

FIG. 13.—Auricular fibrillation, slowed by digitalis.

FIG. 14.—Two months after operation. Normal rhythm. Rate 75.

severe case with marked exophthalmos, tremor, and a pulse-rate of 153. The heart was enlarged, but regular, and there were no murmurs. An X-ray film was taken at 7 feet from the tube to show the size of the heart (Fig. 5), and the transverse diameter of the heart was 16·1 cm. His electrocardiogram is shown in Fig. 7. His operation was done in June, 1928, and five months afterwards the pulse-rate was 109 (Fig. 8), and the transverse diameter of the heart had decreased to 14·7 cm. (Fig. 6.) I saw him again in May, 1930, when his pulse-rate was 88 and the heart was the same size. He had neither exophthalmos nor tremor, and he felt and looked a perfectly fit man.

**Case 4** is one in which I was associated with Mr. C. Ferrier Walters. It was that of a woman who had a toxic adenoma of the thyroid, and rapid, irregular heart characteristic of auricular fibrillation. She complained of shortness of breath, but had no objective signs of congestive heart failure. Digitalis was given till the heart-rate was slowed, and then the adenoma was removed. Three weeks later she was noted to have a regular pulse, and an electrocardiogram showed that her rhythm had become normal. (Figs. 9 and 10.) The operation was in August, 1927; she came up for examination in May, 1930, and her rhythm was found to be still normal. She is no longer short of breath, and is doing all her home work.

**Case 5** was a man of good physique who was sent up by Dr. Bernard because he had been turned down for a good railway job on account of his heart. On examination he was found to have auricular fibrillation with no murmurs, and X-rays showed the transverse diameter of the heart was 15·7 cm. (Fig. 11.) He had a small adenoma of the thyroid with some tremor. He was admitted, after some delay while he was considering the question of operation. When Mr. Short saw him the adenoma was so small that it was hard to perceive, but it was found and operation was agreed upon. The heart was first slowed by digitalis and then the adenoma removed. The fibrillation, however, persisted, so after two months he was given quinidine. This was given in doses of 0·4 grams every two hours, and after the fourth dose his rhythm changed and became regular. After nine days an electrocardiogram was taken. (Figs. 13 and 14.) The pulse-rate was now 75. An X-ray film was taken, and the heart's transverse diameter was 13·5 cm. (Fig. 12.) He was examined again in April, 1930, five months later, and he informed me that he had now passed the doctor, obtained his job, and felt perfectly fit.

We have met with two cases in which glycosuria was present. In one of these there were all the symptoms of diabetes in addition, and ketones were present in the urine. She was given 25 units of insulin, covered with an ounce of glucose, previous to the operation. She did perfectly well, and since the operation the glycosuria has disappeared, with all the other symptoms of diabetes.

The results of operation in these 45 cases have impressed me very much as to the certainty of relief, provided enough of the thyroid is removed during the operation. It is well known that the result of medical treatment is uncertain, and it seems to me to be debatable whether it is fair to submit a case to prolonged treatment by medical means when the issue is in doubt.

One knows that operation will relieve, but of course there is a certain risk during that operation, though the risk is getting less and less, thanks to improvement in the technique of the surgeons. My own impression is that in an early case of Graves' disease a chance should be given to medical treatment, but that if no improvement is shown in six months the question of operation should be seriously considered. In a case of well-established Graves' disease operation should be proceeded with at once. If auricular fibrillation has supervened operation is the more certainly needed, for operation is the only means of removing the factor by which this condition is caused. If this factor be removed there should be no recurrence of the abnormal rhythm, whereas as long as the irregularity persists the patients are condemned to be chronic invalids. In cases where there is an adenoma of the thyroid, this should be removed by operation as soon as symptoms arise.

## Figures and Tables

**Fig. 1 f1:**
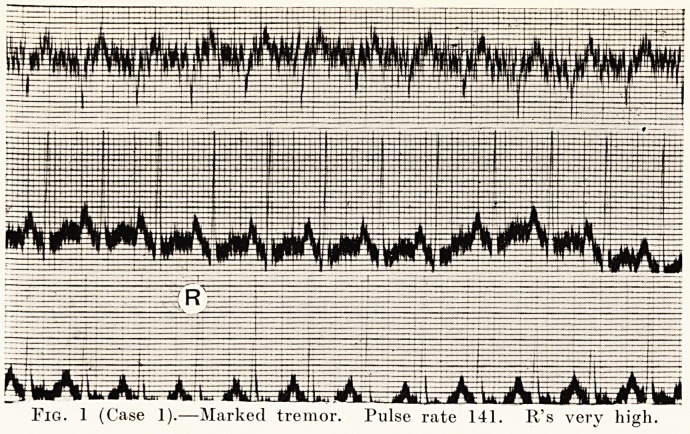


**Fig. 2 f2:**
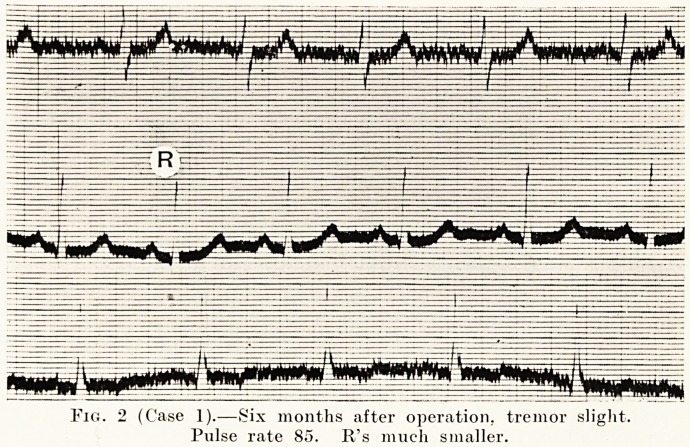


**Fig. 3 f3:**
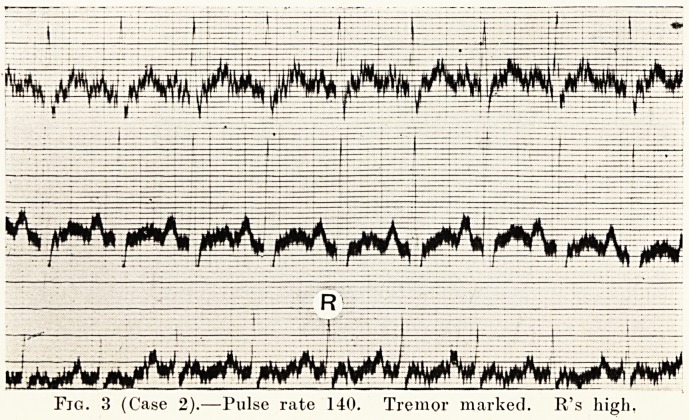


**Fig. 4 f4:**
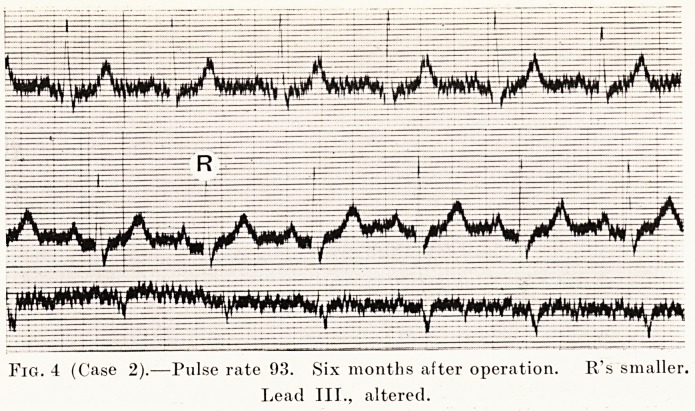


**Fig. 5 f5:**
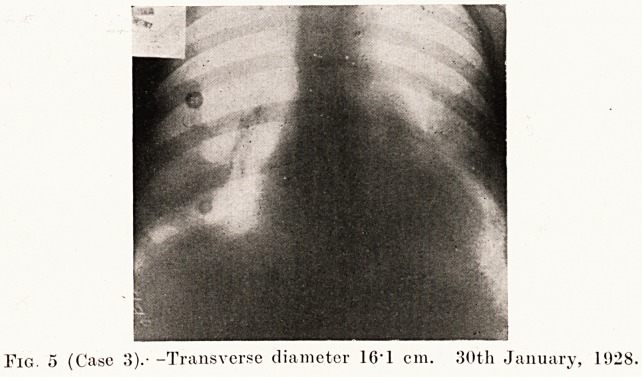


**Fig. 6 f6:**
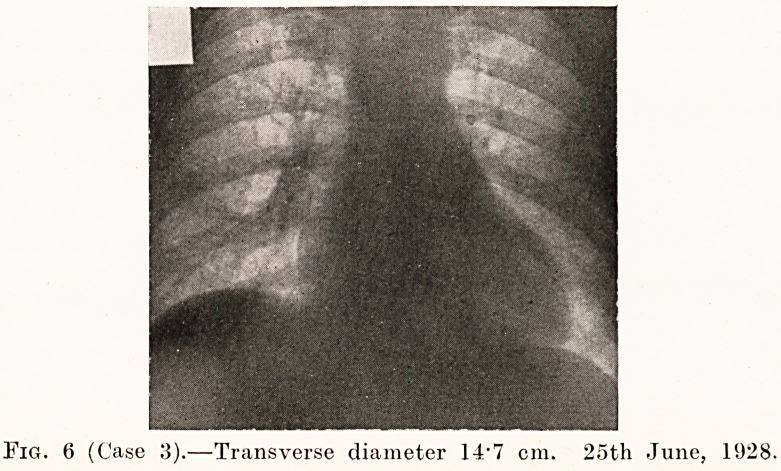


**Fig. 7 f7:**
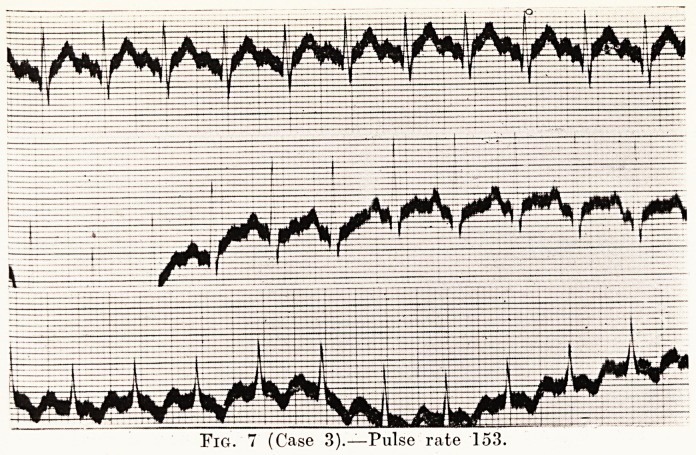


**Fig. 8 f8:**
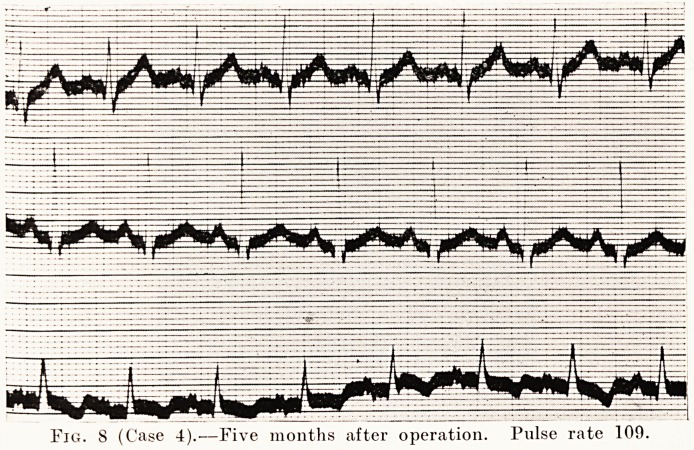


**Fig. 9 f9:**
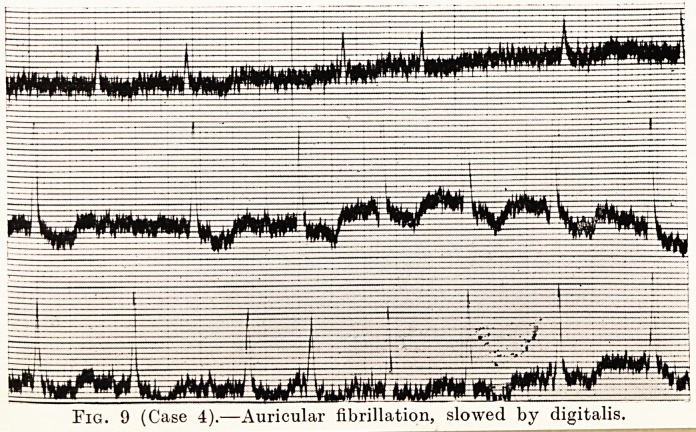


**Fig. 10 f10:**
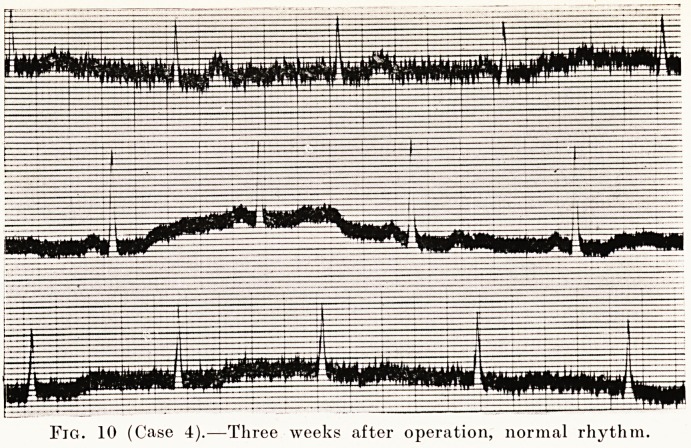


**Fig. 11 f11:**
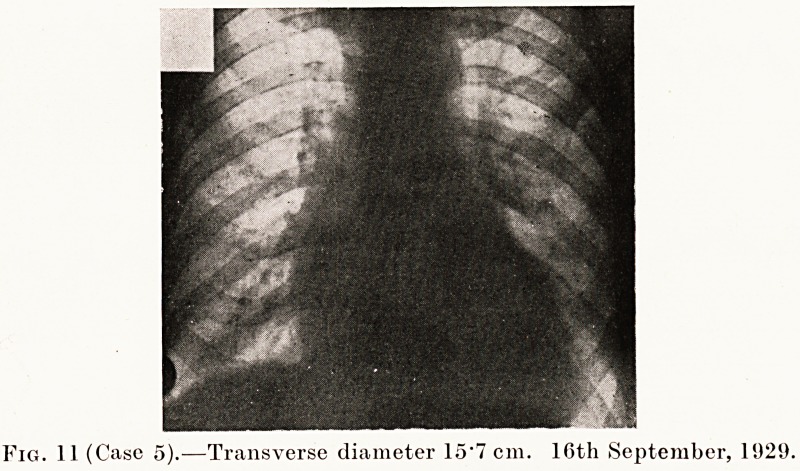


**Fig. 12 f12:**
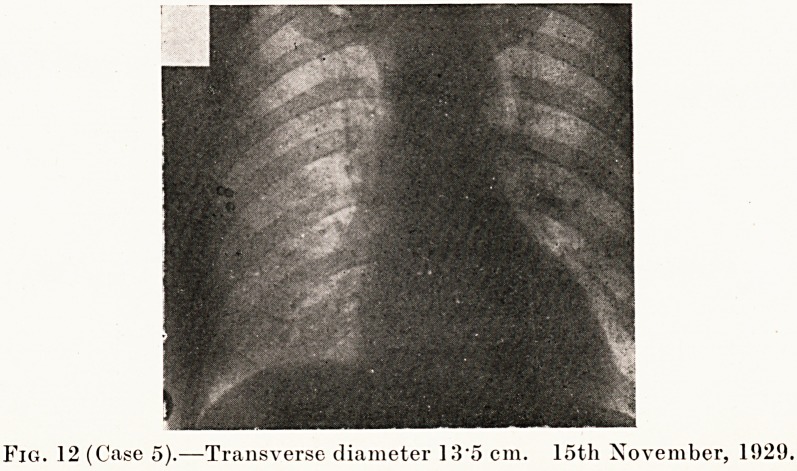


**Fig. 13. f13:**
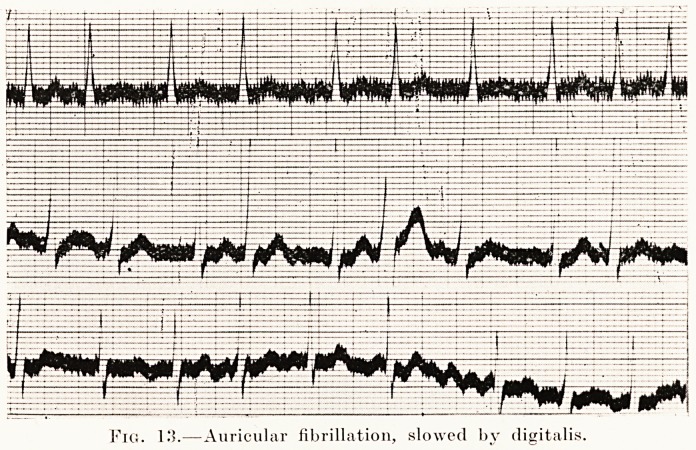


**Fig. 14. f14:**